# Disrupted structural brain networks across psychiatric disorders determined using multivariate graph analyses

**DOI:** 10.1192/j.eurpsy.2023.660

**Published:** 2023-07-19

**Authors:** R. K. Paunova, D. Stoyanov, C. Ramponi, A. Latypova, F. Kherif

**Affiliations:** ^1^Department of Psychiatry and Medical Psychology and Research Institute, Medical University Plovdiv, Plovdiv, Bulgaria; ^2^Department of Clinical Neurosciences, Centre for Research in Neuroscience, CHUV—UNIL, Lausanne, Switzerland

## Abstract

**Introduction:**

Identifying the specific brain pattern characterizing psychiatric disorders could lead us to precise diagnostic process, better treatment plan and outcome prediction. Structural covariance is a graph-analysis method with which disruptions in large scale brain network organization can be observed. More studies, employing this method in psychiatry, are still needed.

**Objectives:**

The current study aims to investigate how the main psychiatric disorders – schizophrenia, major depressive disorder, bipolar disorder, affect brain circuitry by means of multivariate graph theory, more specifically – structural covariance. We hypothesized that specific abnormalities in the brain circuits would be found in separate diagnostic entities.

**Methods:**

164 subjects were included with schizophrenia-SCH (n=17), bipolar disorder-BD(n=25), major depressive disorder–MDD(n=68) and a healthy control group-HC(n=54). Each participant provided a written informed consent and the study protocol was approved by the University’s Ethics Committee. High resolution structural MRI was acquired, and preprocessing was performed using SPM 12 toolbox. The structural covariance method was applied consisting of calculation of the correlation across subjects between the different pairs of regions by using the gray matter average volume. We used the threshold statistic to binarize the covariance matrix and transform it into an adjacency matrix. This allows us to compare psychiatric disorders at a network level by calculating measures such as authorities, hubs and outdegree.

**Results:**

61 statistically significant regions were found for the whole sample. The matrices of the four groups were compared according to their ‘authorities’ ,‘hubs’ and ‘outdegree’ as first, second and third ranking variables, respectively. In the group comparison between HC and BD patients the top five significant regions were Planum temporale (PT), Putamen, Precuneus (PreCu), Calcarine cortex (Calc_cor) and Postcentral gyrus medial segment (PostCGms). The MDD group demonstrated the following regions with most significant difference including Precentral gyrus (PreCG), Entorhinal area (EntA), Amygdala (Amy), Anterior cingulate gyrus (ACC), Anterior insula (AI). While SCH grop was charachterized by ACC, PreCG – medial segment, PostCGms, anterior orbital gyrus, and frontal pole.

**Conclusions:**

The results of our study demonstrated that schizophrenia and mood disorders have specific disturbances in brain network structural organization, affecting hubs of default mode network, salience network, motor, sensory and visual cortex, as well as limbic system. These alterations might elucidate the pathophysiological mechanisms of common symptoms of the disorders under investigation including perceptual, affective and cognitive disturbances.

**Disclosure of Interest:**

None Declared

